# Treatment decision‐making during outpatient clinic visit of patients with esophagogastric cancer. The perspectives of clinicians and patients, a mixed method, multiple case study

**DOI:** 10.1002/cam4.4596

**Published:** 2022-02-15

**Authors:** Josianne C. H. B. M. Luijten, Linda Brom, Pauline A. J. Vissers, Yes. A. J. van de Wouw, Fabienne A. R. M. Warmerdam, Joos Heisterkamp, Stella Mook, Jamal Oulad Hadj, Marc J. van Det, Liesbeth Timmermans, Maarten C. C. M. Hulshof, Hanneke W. M. van Laarhoven, Camiel Rosman, Peter D. Siersema, Marjan J. Westerman, Rob H. A. Verhoeven, Grard A. P. Nieuwenhuijzen

**Affiliations:** ^1^ Department of Research & Development Netherlands Comprehensive Cancer Organization (IKNL) Utrecht The Netherlands; ^2^ Netherlands Association for Palliative Care (PZNL) Utrecht The Netherlands; ^3^ Department of Medical Oncology Viecuri Medical Centre Venlo The Netherlands; ^4^ Department of Medical Oncology Zuyderland Hospital Heerlen The Netherlands; ^5^ Department of Surgery Elisabeth Tweesteden Hospital Tilburg The Netherlands; ^6^ Department of Radiation Oncology University Medical Center Utrecht, Utrecht University Utrecht The Netherlands; ^7^ Department of Medical Oncology Gelre Hospital Apeldoorn The Netherlands; ^8^ Department of Surgery Hospital group Twente Almelo The Netherlands; ^9^ Department of Primary and Community Care Radboud university medical center Nijmegen The Netherlands; ^10^ SPKS Leven met maag‐ of slokdarmkanker Utrecht The Netherlands; ^11^ Department of Radiation Oncology Cancer Center Amsterdam, Amsterdam UMC, University of Amsterdam Amsterdam The Netherlands; ^12^ Department of Medical Oncology Cancer Center Amsterdam, Amsterdam UMC, University of Amsterdam Amsterdam The Netherlands; ^13^ Department of Surgery Radboud University Medical Center Nijmegen The Netherlands; ^14^ Department of Gastroenterology and Hepatology Radboud University Medical Center Nijmegen The Netherlands; ^15^ Department of Epidemiology and Biostatistics Amsterdam UMC Amsterdam The Netherlands; ^16^ Department of Surgery Catharina Hospital Eindhoven The Netherlands

**Keywords:** clinicians' perspectives, esophageal and gastric cancer, multidisciplinary team meeting, patients' perspectives, treatment decision‐making

## Abstract

**Background:**

The probability of undergoing treatment with curative intent according to the hospital of diagnosis varies for esophagogastric cancer in the Netherlands. Little is known about the factors contributing to this variation. This study aimed to improve the understanding of the differences between the multidisciplinary team meeting treatment proposal and the treatment that was actually carried out and to qualitatively investigate the differences in treatment decision‐making after the multidisciplinary team meeting treatment proposal between hospitals.

**Methods:**

To gain an in‐depth understanding of treatment decision‐making, quantitative data (i.e., multidisciplinary team meeting proposal and treatment that was carried out) were collected from the Netherlands Cancer Registry. Changes in the multidisciplinary team meeting proposal and applied treatment comprised changes in the type of treatment option (i.e., curative or palliative, or no change) and were calculated according to the multivariable multilevel probability of undergoing treatment with curative intent (low, middle, and high). Qualitative data were collected from eight hospitals, including observations of 26 outpatient clinic consultations, 30 in‐depth interviews with clinicians, seven focus groups with clinicians, and three focus groups with patients. Clinicians and patients' perspectives were assessed using thematic content analysis.

**Results:**

The multidisciplinary team meeting proposal and applied treatment were concordant in 97% of the cases. Clinicians' implementation of treatment decision‐making in clinical practice varied, which was mentioned by the clinicians to be due to the clinician's personality and values. Differences between clinicians consisted of discussing all treatment options versus only the best fitting treatment option and the extent of discussing the benefits and harms. Most patients aimed to undergo curative treatment regardless of the consequences, since they believed this could prolong their life.

**Conclusion:**

Since changes in the multidisciplinary team meeting‐proposed treatment and actual treatment were rarely observed, this study emphasizes the importance of an adequately formulated multidisciplinary team meeting proposal.

## Graphical abstract

The probability of undergoing treatment with curative intent according to the hospital of diagnosis has been shown to vary considerably for esophagogastric cancer in the Netherlands. Little is known about the factors that contribute to this variation in practice. In this study, we aimed to improve our understanding of the differences between the multidisciplinary team meeting (MDTM) treatment proposal and the treatment that was actually carried out and to qualitatively investigate the differences in treatment decision‐making (TDM) after the MDTM treatment proposal between hospitals.

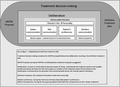

## INTRODUCTION

In the Netherlands, almost 4200 patients are annually diagnosed with esophageal and gastric (esophagogastric) cancer, with over 3000 cancer‐related deaths per year.^1^ Guidelines state that treatment with curative intent should be considered in patients without distant metastasis, depending on the patients' general health status and local extent of disease. The cornerstones of treatment with curative intent for nonmetastatic esophagogastric cancer and early‐stage tumors are resection with or without (neo)adjuvant chemo(radiation) therapy and endoscopic resection, respectively.^2^, ^3^, ^4^, ^5^ For patients with esophageal cancer, definitive chemoradiation is a reasonable alternative with curative intent for frail patients, patients with unresectable locally advanced cancer, or patients who refrain from surgery.^3^ Nevertheless, the probability of undergoing treatment with curative intent has been shown to vary considerably depending on the hospital of diagnosis, with a significant effect on survival in 2010–2013 (hazard ratios of hospitals with a high probability of undergoing treatment with curative intent in esophageal cancer 1.15, 95% confidence interval [CI] 1.07–1.24 and gastric cancer 1.21, 95% CI 1.04–1.41).^6^, ^7^, ^8^

Practice variation can only partially be explained by differences in patient‐ and tumor‐related factors.^6^, ^7^, ^8^ Hence, other factors might explain variation in treatment decisions, such as organization of clinical pathways, the emphasis and subsequent advice of multidisciplinary team meetings, and treatment decision‐making during the outpatient clinic visit after the multidisciplinary team meeting, which encompasses a treatment decision‐making process between physicians and patients.^9^, ^10^, ^11^, ^12^ Thus, the best available evidence of possible outcomes should be shared with the patient, to guide the patient's considerations on treatment preferences.^13^ Hence, one of the aspects that could attribute to practice variation may be a change in the multidisciplinary team meeting proposal caused by this process. Knowledge about these changes could shed light on whether variation in practice between hospitals occurs at the multidisciplinary team meeting level or more during the treatment decision‐making process thereafter in the outpatient clinic.

This study aimed to quantitatively investigate the compliance to the multidisciplinary team meeting proposal when compared to the treatment carried out and to gain insight into factors explaining these changes based on qualitative observations during outpatient clinic visits, interviews with clinicians, and focus groups with clinicians and patients.

## METHODS

This study is part of a mixed methods multiple case study investigating causes of hospital practice variation in the curative treatment of esophagogastric cancer: the VARIATE‐project (Box 1 describes the study design of the VARIATE‐project). The current sub‐study focuses on identifying compliance with the multidisciplinary team meeting treatment proposal, as a result of the treatment decision‐making process during the outpatient clinic visit after the multidisciplinary team meeting combined with quantitative and qualitative data. Quantitative data were used to assess the differences between the multidisciplinary team meeting proposal and actual treatment carried out. Outpatient clinic observations, semi‐structured interviews, and focus groups with clinicians were used to gain insight into the clinicians' perspective on treatment decision‐making, and patient focus groups were organized to gain insight into the patients' perspectives on treatment decision‐making. Clinicians' perspectives included the physicians' convictions and values regarding treatment options, their beliefs and attitudes regarding treatment decision‐making, and the input of a patient's proxy. Patients' perspectives included the patients' experiences regarding treatment decision‐making during the outpatient clinic visit, reasons for selecting a specific treatment modality, their experiences with the discussions with their doctor, and their treatment experiences.

BOX 1 All patients diagnosed with esophageal and gastric cancer in the Netherlands are registered in the Netherlands Cancer Registry (NCR). Previous multivariable multilevel analyses of potentially curable patients diagnosed in the period 2015–2017 have shown that the probability of receiving treatment with curative intent differed according to the hospital of diagnosis.^8^ Hospitals were divided into three tertiles: low, middle, or high probability of undergoing treatment with curative intent. Patients diagnosed in a hospital with a high probability of receiving treatment with curative intent had a significant better long‐term survival.^8^ In order to obtain in‐depth information and knowledge of the underlying mechanisms of hospital practice variation in proposing treatment with curative intent the VARIATE‐ study (VariAtion in the cuRatIve treatment of esophAgeal and gasTric cancEr) was developed, which was financed by the Dutch Cancer Society.

Received treatment with curative intent was defined as endoscopic or surgical resection, initiation of surgery (without resection), definitive chemoradiation (external beam radiotherapy and concurrent chemotherapy; including initiation of chemoradiation). Palliative treatment was defined as: palliative systemic therapy, palliative radiotherapy, and best supportive care.

**Design:**

The VARIATE study is a mixed methods multiple case study, which combines qualitative and quantitative research. A purposive sample^43^ of eight cases (i.e., hospitals) participated. These hospitals were a representative sample of Dutch hospitals regarding the probability of offering treatment with curative intent (low, low, or middle for gastric or esophageal cancer, and high), hospital type, size, and geographical location.

*Recruitment*: Surgeons or medical oncologists from 11 different hospitals were invited by email. After interest was voiced, JL presented the study during the multidisciplinary team meeting (MDTM) of the eight interested hospital to assess the interest of the multidisciplinary team. All hospitals and team members who saw the presentation wished to participate in this study.

This study used an iterative approach for qualitative data collection and analyses, data collection consisted of:

1. Observations of (Upper‐GI specific) MDTMs (2–4 MDTMs per hospital) and outpatient clinic visits (minimum of two outpatient clinic visits per hospital)
2. Semi‐structured interviews (*n* = 30) with clinicians involved in the multidisciplinary care for esophageal and gastric cancer (i.e.*, surgeons* (*S*, *n* = 8), *medical oncologist* (*MO*, *n* = 6), *radiation oncologist* (*RO*, *n* = *5*), *gastroenterologists* (*GE*, *n =* 6), and *case managers* (*CM*, *n* = 5))
3. Focus groups with clinicians in order to validate and further enrich the results of their own hospital (*n* = 7).
4. Focus groups with patients diagnosed with potentially curable esophageal‐ or gastric cancer were organized to explore factors related to their treatment choices (*n* = 3: low, middle, and high probability hospital).

Based on the analysis of the first three hospitals the following decisions regarding the quantitative and qualitative data collection in the further hospitals were made:

1. Depending on the emerging topics from previous interviews the topic list was altered (more focus on: MDTMs, cases of doubt, shared decision‐making).
2. Clinicians in the other five hospitals were selected for interviewing through emergent sampling (i.e., gastroenterologist that did not treat early carcinomas were not invited for participation, recent new members in multidisciplinary teams were not invited for participation).
3. Additional quantitative data for potentially curable patients diagnosed in 2015–2017 was gathered in XX hospitals in the Netherlands (i.e., data were gathered by the NCR regarding diagnostics, the MDTM treatment proposal and outpatient clinic visits) in order to gain insight in clinical pathways and alterations in MDTM treatment proposal.

The VARIATE‐study focusses on the organization of clinical pathways and MDTMs and the outpatient clinic visit.

**Analyses:**

1. Qualitative analyses: Interviews were audio recorded, transcribed per verbatim and summarized (all by JL) and shared with the interviewed clinicians serving as member check. Next, the interviews were reviewed and coded, using open coding as described by Strauss and Corbin.^44^ To minimize subjectivity the first 11 transcripts were independently coded by two researchers (JL, PV) and discussed until consensus was reached.^45^ The remaining 19 transcripts were coded by JL. A summary was written for each interview and each hospital. Using thematic content analyses emerging themes were found.^18^ Simultaneously, through a constant comparison across and within cases, relations were searched for and themes were identied.^19^ The core study group (JL, PV, RV, GN) met weekly to discuss analyses, refine the codebook and identify emerging themes. The coding process was facilitated by Atlas ti 8 software.

Quantitative analyses: Quantitative data was analyzed according to the probability of receiving treatment with curative intent using SAS® version 9.4 (SAS Institute). A *p*‐value below 0.05 was considered statistically significant.

### Data collection procedures

#### Quantitative research

Patients with potentially curable (cT1‐4a or cTx, any cN, cM0) esophagogastric cancer diagnosed between 2015 and 2017 were selected from the nationwide population‐based Netherlands Cancer Registry (NCR). Patients diagnosed with a gastroesophageal junction tumor were included in the esophageal cancer group. Information on patient characteristics, such as sex, age, modified Charlson Comorbidity Index score, and Eastern Cooperative Oncology Group (ECOG) performance status, and tumor characteristics, such as tumor location, histology, stage, and treatment, were collected from medical records by specially trained data managers of the NCR.

Additional data regarding the final multidisciplinary proposed treatment plan prior to the start of treatment were collected from 38 hospitals for patients with esophageal cancer and 68 hospitals for patients with gastric cancer. As the incidence rate of gastric cancer is lower than that of esophageal cancer in the Netherlands, more hospitals (*n* = 68) were included as a representative sample.

Differences between the proposed and actual treatments were assessed, and treatments with curative intent were defined as endoscopic resection (endoscopic mucosal resection or endoscopic submucosal dissection), surgical resection of the primary tumor (with or without [neo]adjuvant chemo[radio]therapy), or definitive chemoradiation (for esophageal cancer). Palliative treatment was defined as systemic therapy only, radiotherapy only, and best supportive care (e.g., stent placement).

#### Qualitative research: Observations, interviews, and focus groups

Eight hospitals were selected based on hospital type (academic resection hospital [*n* = 3], regional resection hospital [*n* = 4], and referring non‐resecting hospital [*n* = 1]), probability of offering treatment with curative intent (low [L] *n* = 2, low/middle [L/M] *n* = 2, and high probability [H], *n* = 4),^8^ and size and geographical location in the Netherlands (see methods regarding hospital selection in Box 1), to achieve a deviant case sampling.^14^ A detailed description regarding the calculation of probability of treatment with curative intent classification has been described previously..^8^

From January 2019 to November 2020, observations during multidisciplinary team meetings and outpatient clinic visits, interviews with clinicians, and focus groups (with clinicians and patients) were conducted. Data were collected and analyzed iteratively. All data were collected by a medical doctor (JL), who was trained to interview, organize focus groups, and analyze the data together with two experienced researchers in the field of qualitative research (LB, MW). Medical oncologists, surgeons, radiation oncologists, gastroenterologists, and case managers (e.g., nurse practitioners, physician assistants) involved in esophagogastric care were all observed and interviewed in the first three hospitals. After the iterative analyses of these three hospitals, emergent themes were discussed within the research team. Thereafter, clinicians in the other five hospitals were selected for interviewing through emergent sampling (see method section Box 1), which implies that sampling decisions were made during data collection as the study progressed.^15^

##### Observations

Twenty‐six outpatient clinic visits in eight hospitals were observed in half‐day sessions (approximately 4 h), focusing on how clinicians and patients dealt with the multidisciplinary team meeting proposal, discussed treatment options, discussed benefits and harms, and clinician‐patient interaction. Field notes were kept during the observations and summarized at the end of each observation. Observations and informal conversations were helpful in building a relationship of trust (rapport) with the clinicians and were used as inputs for the interviews and focus groups.

##### Interviews

Semi‐structured interviews with clinicians were conducted using a topic list (Supplementary 1), based on the expertise of the research team and literature, such as the organization of healthcare and multidisciplinary team meetings^10^, ^11^, ^16^ and physician attitudes toward treatment options.^17^ During all interviews, the opportunity was provided to discuss topics not part of the topic list, exploring new themes that evolved during the interview. Broad topics (Supplementary 1) were discussed during the interviews conducted in the first three hospitals. During the course of this study and through iterative analyses, the topic list evolved, encompassing factors contributing to treatment decision‐making during the outpatient clinic visit, such as the physicians' convictions and values regarding treatment options, their beliefs and attitudes regarding shared decision‐making, and the input of a patient's proxy. These factors led to more focused interviews in the last five hospitals. Semi‐structured interviews were performed by one researcher (JL) with a mean duration of 39 (range 25–54) min. Interviews were audio‐recorded and transcribed ad verbatim (JL). All interviews were summarized. Summaries were sent to each interviewed clinician to assess for accuracy, serving as a member check. All clinicians agreed to the summaries' accuracy.

##### Clinician focus groups

In seven of the eight hospitals, focus groups were conducted after the observations and interviews with 3–4 clinicians. In the included referring hospital, only observations and interviews were conducted, and no focus groups were performed, since only two clinicians were involved in the upper gastrointestinal (GI) clinical pathway. Each focus group started with a presentation of the most important results of the observations and interviews, followed by a discussion in which the clinicians were encouraged to further discuss, complement, or contradict the results of the findings in their hospital. Focus groups were physically organized within the hospital (*n* = 3) or videoconference (*n* = 4) due to the severe acute respiratory syndrome‐coronavirus disease 2019 pandemic and lasted for an average of 1 h and 30 min. The focus groups were observed by one of the two members of the research group (RV, PV) and were all moderated by JL. The focus group moderator and observer discussed all focus groups directly after the focus groups; thereafter, the focus group audio recordings were summarized.

##### Patient focus groups

In three resection hospitals, patients who were diagnosed with a curable tumor stage were recruited for the patient focus group. Thus, patients with a curable tumor stage undergoing palliative treatment were excluded in the focus groups. Patients (*n* = 18) were informed by their treating physician about the VARIATE‐project. After consent was provided, the patients were invited to participate in a focus group in the hospital in which they underwent treatment. All but one invited patient agreed to participate. One patient did not participate due to logistical reasons (see Supplementary 2 for patient demographics).

The focus group guide (Supplementary 3) was used to structure patient's discussion. The focus groups addressed the patients' experiences regarding treatment decision‐making during the outpatient clinic visit, reasons for selecting a specific treatment modality, their experiences with patient‐doctor discussions, and their treatment experiences. Focus groups were organized in a conference room of the hospital and lasted for an average of 1 h and 29 min (range 1 h 15 min–1 h 43 min). Three focus groups were conducted with five to six patients per focus group, moderated by LB, and observed by JL.

### Data analyses

#### Quantitative data analyses

The primary quantitative outcome parameter was the proportion of patients in whom the multidisciplinary team meeting proposal differed from the actual treatment carried out. Secondary outcomes were the proportion of patients discussed during a multidisciplinary team meeting, the type of multidisciplinary team meeting proposal, and received treatment according to the hospitals of diagnosis' probability of undergoing treatment with curative intent.

Patients' baseline characteristics and outcome parameters were reported as frequencies with percentages. To evaluate baseline characteristics and outcome parameters, the chi‐squared test and Fisher exact test were used, when appropriate.

#### Qualitative data analyses

The data used for analyses comprised outpatient clinic field notes, transcripts of the interviews focusing on factors influencing the treatment decision‐making during the outpatient clinic visit, and summaries of the clinician focus groups and patient focus groups. A thematic content analysis was used to identify individual and hospital experiences,^18^ focusing on the clinicians' perceptions and beliefs regarding treatment decision‐making during the outpatient clinic (e.g., *aims, beliefs, personal characteristics, discussed treatment options*) (see Box 1 for a complete overview of the coding process and the identification of emerging themes and subthemes) and was based on the treatment decision‐making model created by Elwyn et al.^13^

For each hospital, a similar thematic map summarizing each theme and subtheme per clinician was created. Through constant comparison within and across cases, associations and deviant cases were identified.^19^ Preliminary conclusions from the thematic map were thoroughly discussed by the core research team (JL, PV, RV, GN, MW). The thematic map comprised the themes and interrelations between the codes and themes, such as discussed treatment options, influence of patient characteristics, and influence of clinician's personality traits. Thereafter, the themes were discussed with two experts in the field of shared decision‐making (LB and LT).

### Ethics

Ethics approval was not required according to the Medical Research Ethics Committees United of the Netherlands (number: W.18.166). All participating hospitals approved this study. All quantitative data collected by the NCR were de‐identified and coded. Written informed consent was obtained from all participants prior to the interview or start of the patient focus groups. Privacy and confidentiality were protected through pseudonymization. The transcripts and summaries will be stored pseudonymized for a minimum of 10 years, on the secured network of IKNL, to which only the core research team members have access. This study was funded by the Dutch Cancer Society (project number: 10895).

## RESULTS

### Quantitative results: Changes in the multidisciplinary team meeting proposal

Table 1 shows patient characteristics according to the hospital's diagnosis probability of undergoing treatment with curative intent. For esophageal cancer, no significant differences were observed between the probability groups; however, for gastric cancer, significant differences were observed in the number of comorbidities, ECOG performance status, and cT tumor stage. Surgery with curative intent was proposed during multidisciplinary team meeting in 63%, 65%, and 72% of patients diagnosed with esophageal cancer in a hospital with a low, middle, or high probability of undergoing treatment with curative intent, respectively. For gastric cancer, the rates were 75%, 82%, and 82%, respectively. Definitive chemoradiation was proposed during the multidisciplinary team meeting in 13%, 12%, and 15% of patients diagnosed with esophageal cancer in a hospital with a low, middle, or high probability of undergoing treatment with curative intent, respectively. In hospitals with a low, middle, or high probability of proposing treatment with curative intent, the proposed treatment plan for patients with esophageal cancer changed by 5%, 4%, and 3% (*p* = 0.24), respectively, and for gastric cancer, these were 1%, 0%, and 0% (*p* = 0.18), respectively (Table 2). Changes in the multidisciplinary team meeting proposal and actual treatment carried out comprised changes in the type of curative treatment option, such as a surgical proposal changing to definitive chemoradiation (*n* = 42). Nonetheless, no changes from curative intent multidisciplinary team meeting proposals to palliative treatment proposals or vice versa were observed (Table 2). Of the patients diagnosed with esophageal cancer in which the treatment plan was known (*n* = 1293), neoadjuvant treatment followed by resection was proposed in 65% of the patients. Of them, 18% did not undergo the proposed treatment plan: 12% underwent primary surgery, 5% underwent definitive chemoradiation, and 1% underwent endoscopic resection. Of the patients diagnosed with gastric cancer in which the treatment plan was known (*n* = 935), neoadjuvant treatment followed by surgery was proposed in 51% of the patients. Of them, 17% did not receive chemotherapy but directly underwent surgery. Of the patients in which perioperative treatment followed by resection was proposed, 83% underwent neoadjuvant treatment followed by resection of whom 39% did not undergo the adjuvant component. Direct surgery was proposed in 28% of the patients diagnosed with gastric cancer of which 98% underwent surgery (Table 3).

**TABLE 1:** Patient characteristics esophageal and gastric cancer patients according to the hospital of diagnosis low, middle, or high probability of receiving treatment with curative intent

	Esophageal cancer							Gastric cancer						
	Probability													
	Low		Middle		High		*p* value	Low		Middle		High		*p* value
ALL	477	100%	548	100%	625	100%		507	100%	327	100%	545	100%	
Sex							0.75							0.53
Female	136	29%	147	27%	179	29%		199	39%	122	37%	224	41%	
Male	341	71%	401	73%	446	71%		308	61%	205	63%	321	59%	
Age							0.09							0.72
60–74	239	50%	287	52%	340	54%		169	33%	122	37%	195	36%	
<60	100	21%	83	15%	111	18%		76	15%	46	14%	71	13%	
GE 75	138	29%	178	32%	174	28%		262	52%	159	49%	279	51%	
cT Classification							0.59							0.03
cT1	28	6%	35	6%	34	5%		21	4%	12	4%	29	5%	
cT2	133	28%	143	26%	197	32%		202	40%	117	36%	190	35%	
cT3	239	50%	273	50%	305	49%		130	26%	66	20%	117	21%	
cT4	6	1%	10	2%	10	2%		16	3%	20	6%	19	3%	
cTX	71	15%	87	16%	79	13%		138	27%	112	34%	190	35%	
cN Classification							0.22							0.7
cN0	176	37%	211	39%	272	44%		275	54%	182	56%	317	58%	
cN+	260	55%	288	53%	303	48%		161	32%	99	30%	151	28%	
cNX	41	9%	49	9%	50	8%		71	14%	46	14%	77	14%	
Histology							0.07							0.475
Adenocarcinoma	348	73%	386	70%	477	76%		491	97%	321	98%	532	98%	
Squamous cell carcinoma	123	26%	145	26%	134	21%		NA		NA		NA		
Not otherwise specified	6	1%	17	3%	14	2%		16	3%	6	2%	13	2%	
Number of Comorbidities							0.83							0.01
0 comorbidities	192	40%	213	39%	259	41%		166	33%	127	39%	219	40%	
1 comorbidity	151	32%	184	34%	204	33%		187	37%	111	34%	153	28%	
2 or more	116	24%	117	21%	141	23%		135	27%	74	23%	150	28%	
Unknown	18	4%	34	6%	21	3%		19	4%	15	5%	23	4%	
ECOG performance status							0.53							0.001
ECOG 0 and 1	319	67%	368	67%	415	66%		260	51%	141	43%	267	49%	
ECOG 2	39	8%	53	10%	43	7%		37	7%	24	7%	51	9%	
ECOG 3 and 4	16	3%	16	3%	17	3%		31	6%	10	3%	12	2%	
Unknown ECOG	103	22%	111	20%	150	24%		179	35%	152	46%	215	39%	

**TABLE 2:** Treatment and treatment plan in patients diagnosed with esophageal‐ and gastric cancer according to the probability of receiving treatment with curative intent

	Esophageal cancer							Gastric cancer						
	Low probability		Middle probability		High probability		*p* value	Low probability		Middle probability		High probability		*p* value
Total	477	100%	548	100%	625	100%		507	100%	327	100%	545	100%	
Discussed in multidisciplinary team meeting	414	87%	486	89%	547	88%	0.645	411	81%	269	82%	457	84%	0.434
Involvement expert center	407	85%	429	78%	535	86%	<0.0001	362	71%	213	65%	389	71%	0.02
Treatment plan known	383	80%	420	77%	490	78%		343	67%	220	67%	372	68%	
Treatment plan							0.001							0.217^a^
Endoscopic resection	21	5%	13	3%	8	2%		7	2%	3	1%	10	2%	
Resection	240	63%	274	65%	351	72%		258	75%	180	82%	304	82%	
Definitive chemoradiation	51	13%	50	12%	72	15%								
Palliative	58	15%	67	16%	53	11%		37	11%	18	8%	24	6%	
Best supportive care	13	4%	16	4%	6	1%		41	12%	19	9%	34	9%	
Treatment							<0.0001^a^							<0.0001^a^
Endoscopic resection	17	4%	10	2%	9	2%		7	2%	3	1%	7	2%	
Neoadjuvant and/or resection	229	60%	263	63%	341	70%		258	75%	180	82%	307	83%	
Definitive chemoradiation	66	17%	64	15%	81	17%								
Palliative systemic therapy	6	2%	2	1%	5	1%		5	1%	4	2%	4	1%	
Palliative radiation therapy	34	9%	46	11%	29	6%		21	6%	4	2%	8	2%	
Best supportive care	31	8%	35	8%	25	5%		52	15%	29	13%	46	12%	
Alteration in treatment plan	18	5%	18	4%	13	3%	0.24	2	1%	0	0%	0	0%	0.18^a^
Endoscopic resection → surgical resection¥	1	5%												
Surgical resection → endoscopic resection	0		0		2	15%		2	100%					
Surgical resection → definitive chemoradiation	16	89%	16	89%	10	77%								
Definitive chemoradiation → surgery	1	5%	2	11%	1	8%								

*Note*: The treatment plan is solely assessed of patients discussed in a multidisciplinary team meeting prior to treatment. Treatment plan, treatment, and alteration in treatment plan were solely assessed if the treatment plan was known.

a For groups with numbers <5 Fishers exact test was used.

**TABLE 3:** Multidisciplinary team meeting (MDTM) proposed treatment plan versus actual received treatment

Esophageal cancer						Gastric cancer					
MDTM proposal (*n* = 1293)			Received treatment			MDTM proposal (*n* = 935)			Received treatment		
Endoscopic resection	42	3%	Endoscopic resection	34	81%	Endoscopic resection	20	2%	Endoscopic resection	15	75%
			Neoadjuvant treatment followed by resection	7	17%				Neoadjuvant treatment followed by resection	3	15%
			Primary resection	1	2%				Primary resection	2	10%
Neoadjuvant treatment followed by resection	841	65%	Neoadjuvant treatment followed by resection	697	82%	Neoadjuvant treatment followed by resection	474	51%	Neoadjuvant treatment followed by resection	393	83%
			Primary resection	100	12%				Primary resection	81	17%
			Endoscopic resection	2	1%						
			Definitive chemoradiation	42	5%						
Primary resection	24	2%	Primary resection	24	100%	Primary resection	266	28%	Primary resection	261	98%
									Neoadjuvant treatment followed by resection	3	1%
Definitive chemoradiation	173	14%	Definitive chemoradiation	169	97%				Endoscopic resection	2	1%
			Neoadjuvant treatment followed by resection	2	1.5%						
			Primary resection	2	1.5%						
Palliative treatment	213	16%	Palliative treatment	213	100%	Palliative treatment	175	19%	Palliative treatment	173	99%
									Primary resection	2	1%
Alterations in treatment plan				156	12%					93	10%

### Qualitative results: From treatment proposal to the actual treatment carried out

**FIGURE 1:**
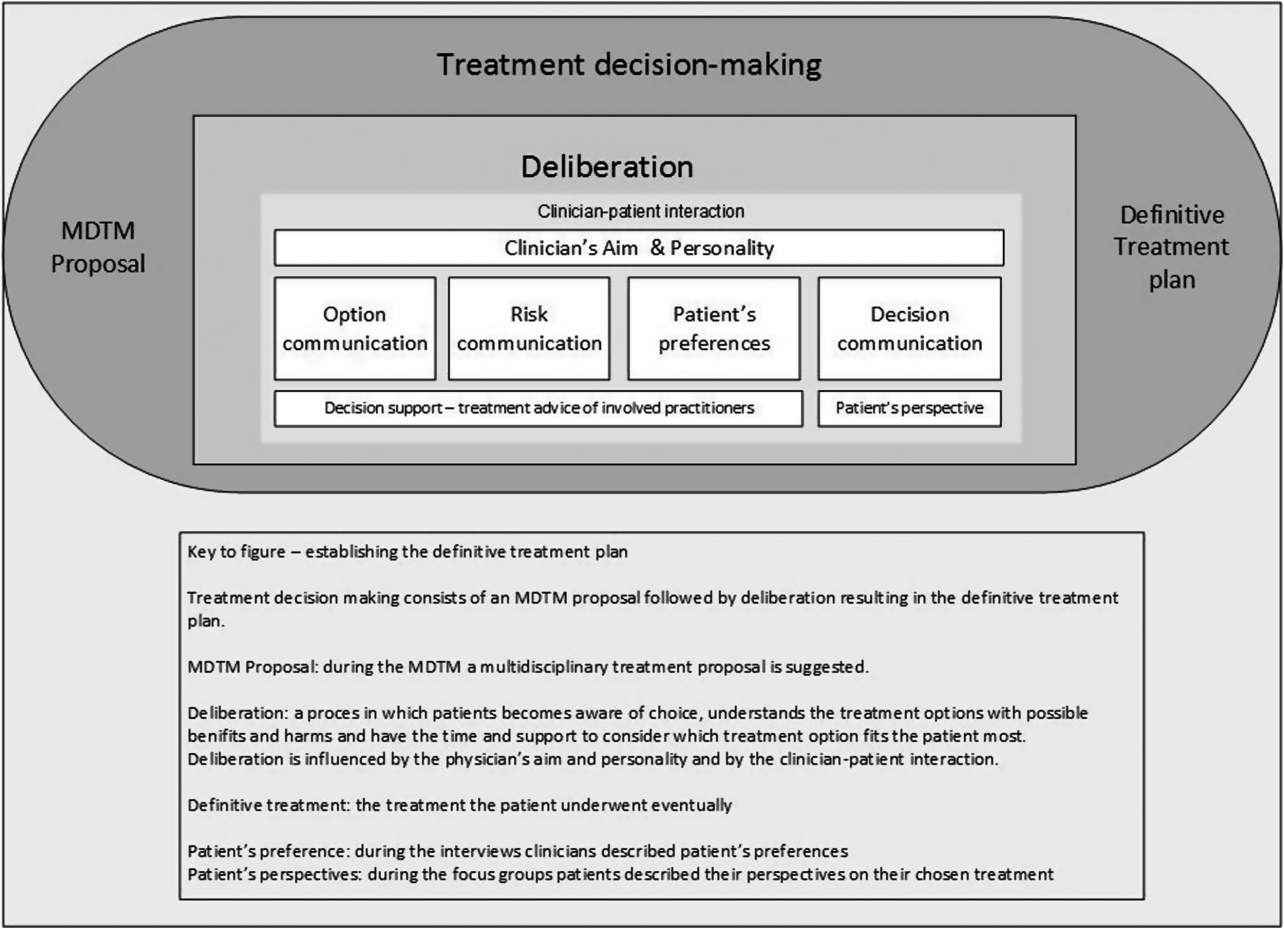
Treatment decision‐making

Figure 1 shows a visualization of the treatment decision‐making process during the outpatient clinic visit, in which the multidisciplinary team meeting proposal was discussed with the patient. In this process, the patient should become aware of having a choice, understand the treatment options (option communication) with the possible benefits and harms (risk communication), and feel that there is sufficient time and support to consider which treatment option fits most to the patients' preferences (shared decision‐support), resulting in decision communication (treatment decision‐making). A comprehensive description of the factors that play a role in treatment decision‐making during the clinician‐patient interaction and deliberation is shown in Table 4, such as clinician's aims, personality, and conversation style. These themes and subthemes are described in more detail below and complemented by the patient's perspectives on treatment decision‐making, which were expressed during the focus groups (Table 5 displays quotes of the patient focus groups).

**TABLE 4:** Treatment decision‐making during the outpatient clinic visit: a clinician's perspective

Theme	Subtheme	Category
Discussing the multidisciplinary team meeting proposal with the patient	Context	Setting and participants (e.g., *multidisciplinary outpatient clinic visit vs. monodisciplinary outpatient clinic visit, referring clinician vs. treating clinician discusses treatment proposal with patient*)
	Clinician's aims	Accomplishing informed consent (e.g.*, following the Medical Treatment Contracts Act [WGBO], taking into account the patient's ability to reason, considered and broadly carried decision)* Providing tailored information (e.g.*, providing patient with realistic perspective, outlining context and uncertainties, adjusting perspective of patients who are misinformed by the referral center)*
	Conversation style	Physician's attitude (e.g., *providing sufficient time and allowing the patients to tell their stories, providing the feeling that the patients and their families are heard; decision should be made together with the patient and their family*) Relationship of trust (e.g., *trust between physician and patient, trust that best decision has been made*) Communication style (e.g.*, jargon‐free communication; conversation flows better in patients diagnosed with multimorbidity since their perspective is more realistic*)
Deliberation	Personal practice style	Personal communication style (e.g., *decisive vs. elaborative, physician showing his/her vulnerability in cases of doubt, empathetic, humor*) Personality (e.g., *providing hope, prepared to give the treatment a chance, daring to propose experimental treatment options, daring to let the patient make the treatment decision*) Personal treatment believes (e.g., *the feasibility of a certain treatment, primum non nocere – first do no harm*)
	Discussion about treatment options	Option communication *Explaining the treatment advice and other options, such as golden standard, primary surgery, definitive chemoradiation, palliative treatment, refraining from treatment* *Explaining that there is always a choice* *Explaining other treatment options only if the patient asks for them* *Explaining how multimorbidity and motivation can have an effect on different treatment options* Risk communication *Explaining benefits and harms, such as postoperative complications and effects on quality of life* *Expectations of treatment effect, such as clear presentation of uncertainty, downstaging of tumor and evaluation* *Explaining treatment burden and discussing the patient's ability to cope with treatment consequences* Patient's preferences *Patient's desires, preferences, and religious values* *Patient's views on their problems and comprehension* *Patient's family and home situation* Decision‐communication *No guidance, such as awaiting the patient's reaction* *Guiding the patient* (i.e.*, toward multidisciplinary team meeting advice or elderly patient toward definitive chemoradiation)* *Trying to determine together with the patient which treatment fits the patient best* *Coping with the patient's persuasion of a certain treatment* *Providing time for consideration* Decision‐support *Advise to the patient to consult other clinicians, such as the general practitioner or medical oncologist* *Patient should have discussed the treatment advice with all involved clinicians prior to decision‐making*

Abbreviation: SES, socioeconomic status.

**TABLE 5:** Treatment decision‐making during the outpatient clinic visit: a patient's perspective

Theme	Subtheme	Category	Patient quotes
Conversation about treatment decision‐making	Patient's experience regarding option communication	Physician input regarding discussed treatment options	“In my case, definitive chemoradiation was not discussed” FG2
			“For a little while, I was in doubt, since the radiation oncologist explained the option of chemoradiation without surgery; however, we had already chosen for the treatment including surgery” FG3
			“There was the possibility to ask questions, but there was no room in the treatment protocol, so once I had the appointment with the surgeon, it was we are going to do this and that and operate” FG2
			“They said you have got one of the most aggressive forms of cancer; in our opinion, surgery is your best option, so I just went along with that” FG3
			“There was no attention for my spouse; nevertheless, they also have to deal with the disease” FG2
		Various media input regarding treatment options and consequences	“Prior to the outpatient clinic visit, I have read about the treatment options and complications on the internet” FG3
		Information load	“A lot of information is discussed in a short time, so you have to filter; I did not remember everything they told me” FG3
			“You are in a kind of whirlwind, but at the end of the day, you know where you stand” FG3
			“It is better if you bring family members to the outpatient clinic consult, so you can discuss the options afterward” FG2
	Patient's experiences regarding risk communication	Not concerned about the effect of harms	“You know that problems can occur, they told you several, but at that time, you are not concerned about them” FG2
			“Let happen what needs to be done” FG1
Treatment decision‐making	Patient's preferences influencing treatment decision‐making	Patient's desire for quality of life	“I have said, from the beginning, no surgery. All the doctors wanted to know why I did not wanted surgery; I believe that quality of life is more important than the duration of my life; then I have to add; I am single and I do not have a safety net” FG3
		Patients hoping for cure	“I do not have a choice, I just want to be able to walk the earth for a very long time” FG1
			“You trust the physician completely, they propose a treatment plan, you want to be cured, so you go for it” FG3
		Patient's trust in physician	“You just give your life in the hands of the other, the one, that will try to fix it” FG1
			“At that time you are not fully accountable; all you can think about is how can I get rid of this […]; then you have to trust the physician” FG3

Abbreviation: FG, focus group.

### Discussing the multidisciplinary team meeting proposal with the patient

#### Context

The setting and participants during the outpatient clinic visit differed between hospitals; for example, some hospitals organized an outpatient clinic visit directly after the multidisciplinary team meeting involving the whole multidisciplinary team, as opposed to other hospitals where patients were seen separately by different clinicians on different occasions after the multidisciplinary team meeting. Additionally, in some hospitals, the referring clinician discussed the multidisciplinary team meeting proposal with the patient, whereas in other hospitals, an upper‐GI involved clinician from the expert center discussed the multidisciplinary team meeting proposal with the patient.

### Clinician‐patient interaction

#### Clinician aims and conversation style

Most frequently, a clinician's intention was to accomplish an informed treatment decision by explaining how the multidisciplinary team meeting decision was substantiated, facilitating the patient to make a balanced decision during treatment decision‐making as explained by a surgeon: “If he understands and comprehends what is happening, and is treatable, also in the postoperative phase (i.e., no mental impairment leading to declining nasogastric feeding), then we would perform surgery” (surgeon 3, low/middle probability). Additionally, most clinicians believed that the treatment decision should be made together with the patient and family members, in which they experience respect and trust that the best treatment decision has been made: “I would like the patient and the family to have the feeling that they are heard and that they understand that we are on the same page regarding the treatment aims” (surgeon 1, low probability). Hence, clinicians believed that it is important that the patient feels that sufficient time is available in which the patients is allowed to discuss their arguments. Clinicians believed that the trust between them and the patient was important. Patients mentioned that they trusted to put their lives into the clinician's hands, and since their will to survive prevailed, they agreed with the proposed (curative) treatment proposal.

The observations showed that communication styles varied among clinicians, such as elaborating and discussing treatment and complications in more detail, which was also explained by a case manager during an interview: “They definitely differ; one of them is more short but decisive, and the other elaborates more; however, their aims are similar” (case manager 8, low/middle probability). Empathy or humor was helpful in putting the patient at ease, as explained by other clinicians.

### Deliberation

#### Clinician's personality and practice style

The clinician's practice style, communication style, and treatment belief are all part of a clinician's personality, which differed between clinicians and was mentioned to affect discussion. For instance, some clinicians were more inclined to guide the patient toward the multidisciplinary team meeting‐proposed treatment plan, whereas others provided more room to let the patient make a treatment decision. According to a gastroenterologist, clinicians should express their vulnerability when discussing uncertainties, as this would benefit the patient's decision‐making: “Naturally, at times, patients do not know which choice to make, and at these times perhaps, we should take more responsibility; a lot of doctors are unsure to discuss their uncertainties with patients about choice A or choice B, since they hold the belief that the doctor should provide the patient with a superior choice” (gastroenterologist 3, low/middle probability).

#### Option communication

Most clinicians explained that all treatment options, including the multidisciplinary team meeting proposal, should be discussed during treatment option communication, as opposed to others who mentioned that they only explain other treatment options if the patient asked for them: “Only if patients ask for it, then we discuss definitive chemoradiation, but we do tell them that it does not provide the best odds” (case manager 2, high probability). Moreover, in the patient focus groups, patients mentioned that not all treatment options were discussed. Clinicians differed in their discussion of the multidisciplinary team meeting proposal during the outpatient clinic. Based on the observations and interviews, some clinicians only discussed the multidisciplinary team meeting proposal, as opposed to others who discussed their personal treatment beliefs in addition to the multidisciplinary team meeting proposal: “Well, that's the question, ultimately, the multidisciplinary team meeting proposal is only an advice; you can deviate from this advice as treating physician, but you have to motivate your decision […], so if the multidisciplinary team meeting's advice is on the conservative spectrum, and I personally belief a more invasive treatment option is feasible, ‘I would like to discuss this with you and then we can make a shared decision’” (surgeon 6, high probability). During treatment decision‐making in the outpatient clinic visit, the clinician's personal treatment beliefs and values played a role according to a surgeon: “The person's ethics, the beliefs of the clinician, are important in how that person comes to a decision; perhaps, life experiences play a role as well” (surgeon 2, high probability).

Most clinicians mentioned that providing tailored information is important, such as providing the patient with a realistic perspective: “Occasionally, patients imagine total alopecia due to chemotherapy; that is however no longer the case in patients treated with chemotherapy for esophageal cancer.” (case manager 1, low probability). Most clinicians aimed to outline the context, such as the effect of patient age on treatment outcome and the uncertainties associated with certain (experimental) treatment options.

#### Risk communication

Most clinicians mentioned that they explain the benefits and harms of the treatment options during risk communication: “In all openness, the benefits and harms are discussed with the patient, weighing what the best treatment option is for this individual patient” (medical oncologist 3, low/middle probability). However, it was observed that the extent of discussing the benefits and harms of the proposed treatment varied among clinicians. Some clinicians only mentioned the probability of postoperative complications, whereas others discussed complications in more detail. Most patients mentioned that they were not concerned about the impact or harms, since they had a strong will to be cured.

#### Patient's preferences

During the patient focus groups, most patients mentioned that they did not experience that there was a choice, since their will to be cured prevailed. Some clinicians mentioned exceptions, for instance, patients who felt that they had a complete life or did not want to compromise their quality of life standards by surgery. This was also explained by one of the patients, stressing that preserving quality of life was more important than prolonging life, which made her choose for definitive chemoradiation. Patients' motivation, preferences, home situation, and family opinions were taken into account during treatment decision‐making according to most clinicians: “I think that we take the interest of the patient into account, or the patient's opinion, what does the patient want, and I can imagine that there are hospitals that are more guiding” (surgeon 8, low/middle probability). Some clinicians explained that they always stress that the patient always has a choice: “I always try to tell them, “yes, you do have a choice” […] I always discuss that, and that we can also refrain from treatment” [medical oncologist 5, high probability). However, most clinicians explained that refraining from treatment was rarely an option for patients.

#### Decision‐communication

Most clinicians recognized their ability to influence the patient's treatment decision‐making during decision‐communication: “It is not like I tell the patient to make a choice, as choosing your food at the Chinese restaurant […], yet I realize that we are able to guide the patient's treatment decision‐making […] it's not only explaining the options, but you can also influence the patient's decision” (surgeon 1, low probability). Based on the observations, clinicians used different techniques, ranging from describing all treatment options without the treating physician's personal preference to guiding the patient to the definitive treatment plan, which was also mentioned by a surgeon: “I try to get an impression how the patient responds to all the treatment options, how they consider the treatment options, or whether they did their own search on the internet” (surgeon 8, low/middle probability). Some clinicians felt that doctors need a certain maturity to guide the patient properly during treatment decision‐making: “It is important that doctors have a certain maturity, a certain confidence, that during uncertainties, they can guide the patient” (gastroenterologist 3, low/middle probability).

#### Decision‐support

During the focus groups, patients mentioned that they needed time to process the discussed treatment options and their own preferences and were not able to decide during the outpatient clinic visit, which was also mentioned by some clinicians. One patient mentioned that he received a significant amount of information during the visit and needed time to think about what was explained during the consultation. In these situations, the clinician organized a second appointment to support the patient in the treatment decision‐making or involved the patient's general practitioner. Most clinicians felt that the patient should discuss the treatment proposal with all involved clinicians prior to a definitive treatment choice is made: “I insist that the patient has also a consultation with the surgeon prior to the start of treatment; this was however not always the case in the beginning” (radiation oncologist 6, high probability). Based on these observations, patients rarely seek contact with peers or provide pamphlets to explain treatment options.

## DISCUSSION

In this mixed method multiple case study, the quantitative results demonstrated that differences between the multidisciplinary team meeting proposal and applied treatment rarely occurred. Despite the qualitative finding that most clinicians held similar opinions regarding the decisional process, the implementation of treatment decision‐making in practice varied. Based on the observations, differences comprised discussing all treatment options versus only the best fitting treatment option and the thoroughness of discussing the benefits and harms of treatment options. Most patients aimed to undergo treatment with curative intent and were less concerned about the possible harms.

Based on the principle that multidisciplinary case discussions lead to improved treatment recommendations, multidisciplinary team meetings have been widely implemented in cancer care. The multidisciplinary team meeting structure enhances communication, decision‐making, and coordination, based on evidence‐based and updated knowledge or expert opinions.^20^ The quantitative results of the present study indicate that changes in the multidisciplinary team meeting proposal and the actual treatment decision during the outpatient clinic visit only rarely occurred. Hence, variation in the probability of undergoing treatment with curative intent between hospitals mainly originates during clinical decision‐making during the multidisciplinary team meeting. Furthermore, it can be hypothesized that the multidisciplinary tumor board generally proposes an adequate treatment plan; thus, sufficient information is available during the multidisciplinary team meeting, including patient characteristics, such as the patient's opinion, resulting in a fitting treatment proposal that matches the individual needs of a specific patient. Therefore, based on the results of the current study, adequate organization of a multidisciplinary team meeting and clinical decision‐making during a multidisciplinary team meeting is invaluable, since most clinicians mentioned that they value the upper‐GI multidisciplinary team meeting treatment proposal and communicate this during the outpatient clinic visit as a treatment advice. Due to the development of multidisciplinary team meetings, the process of individual physician decision‐making has shifted to multidisciplinary decision‐making, yet the definitive treatment decision is a shared decision between the clinician and patient made during the outpatient clinic visit.

In 1956, three basic models of the physician‐patient relationship were described (i.e., activity‐passivity, guidance‐cooperation, and mutual participation).^21^ Recently, treatment decision‐making should consist of mutual participation, in which the physician and patient work together as partners toward a mutually agreed treatment plan. First, awareness of equipoise should be created (explaining that there is no best choice), followed by discussing the benefits and harms. Subsequently, the patient's concerns and preferences should be elicited, and the patient should be supported in the process of deliberation.^22^ The process of treatment decision‐making during the outpatient clinic visit is pivotal, and regardless of the multidisciplinary team meeting proposal, the discussion toward a definitive treatment plan should be personalized to accompany the patient in the treatment decision‐making. During the patient focus groups, it became clear that most patients trusted their clinician in the treatment advice and their strong intention to be cured prevailed, which resulted in following the treatment proposal. In line with a previous study,^23^ the current study demonstrated that clinicians expressed that during treatment decision‐making, a partnership between the clinician and patient in which the patient feels supported and encouraged is important. Furthermore, clinicians explained the importance of the patient having the feeling that they were heard, and that the best treatment decision was made as a joined venture. Importantly, previous studies have demonstrated that greater patient participation during decision‐making has a positive effect on patient satisfaction, coping, and treatment adherence.^24^, ^25^, ^26^ Hence, although the multidisciplinary team meeting treatment proposal only rarely changed during the outpatient clinic visit, treatment decision‐making is an important process in treatment decision‐making that enhances patient empowerment and possibly results in increased patient satisfaction and treatment adherence.

During the interviews, it became apparent that most clinicians recognized their ability to influence patients' decision‐making during decision‐communication. In addition, differences were observed, such as describing all treatment options, including personal preferences, and guiding the patient to the multidisciplinary team meeting proposal or offering treatment options that differed from the multidisciplinary team meeting proposal. In line with previous studies,^27^, ^28^ the qualitative results of the present study demonstrate that the clinician's personality, personal beliefs, and values on treatment (subjective considerations) influence treatment decision‐making during the process of clinical decision‐making during outpatient clinic visits. In 1984, Wennberg reported that subjective physician considerations play a decisive role during clinical treatment decision‐making (i.e., the practice style factor).^29^ He found that physicians held different opinions regarding the best therapy, and that this was unrelated to scientific controversies.^29^ More recent literature has described that the practice style factor reflects a deeply rooted behavioral mechanism regarding convictions of the individual clinician with respect to how to practice medicine,^30^ possibly attributed to the observed differences in offering guidance or withholding a personal opinion in the current study, which is in concordance with a previous study.^31^ Furthermore, variation in practice was observed regarding the discussed treatment options and the extent of discussing benefits and harms during the outpatient clinic visit. The frequent absence of addressing alternative options or refraining from treatment and differences in the extensiveness of discussing potential benefits and harms have also been described in previous studies.^23^, ^32^, ^33^ A potential explanation for this phenomenon may be that clinicians might feel that fostering hope may contribute to the patient's well‐being,^33^ which might explain the finding that clinicians refrained from discussing alternative treatment options with a lower likelihood of survival. Another suggested explanation is that the therapeutic relationship might be damaged by a clinician sharing information on gaps in scientific knowledge, uncertainties, and controversies.^34^ This means that the process of treatment decision‐making might be influenced by the individual practice style factor but yet rarely leads to changes in the multidisciplinary team meeting treatment proposal.

### Strengths and limitations

The main strength of this study is the combination of quantitative data and the clinicians' and patients' perspectives, providing a complete understanding of differences between clinicians toward treatment decision‐making during outpatient clinic visits. Furthermore, the reliability and validity of the data increased due to data triangulation and member checking.^35^, ^36^ Nevertheless, this study has some limitations when interpreting the results. For instance, our findings based on the patient focus groups should be interpreted with caution, since only patients who underwent treatment with curative intent were included. It would have been of added value when patients diagnosed with a curable stage, but who refrained from treatment with curative intent, would have been included. However, we were unable to recruit the patient group. Nevertheless, the outcomes of this study provide insight into the perspectives of patients who underwent treatment with curative intent and their reasoning. Additionally, all observations and interviews were performed by one researcher; nonetheless, potential researcher bias was tried to be prevented during data collection and analyses by peer debriefing.^37^ Furthermore, the last multidisciplinary team meeting prior to treatment was defined as the multidisciplinary team meeting treatment proposal in the quantitative analyses. Since multiple multidisciplinary team meetings were held in some patients, the patient's feedback on previously discussed treatment options might be used as input during the multidisciplinary team meeting in which the multidisciplinary team meeting proposal is finalized. This means that during multidisciplinary team meeting, information regarding the patient's preference might be known and taken into consideration. In addition, at times, an “if then” treatment decision was made during the multidisciplinary team meeting, and since the data managers were only able to formulate one multidisciplinary team meeting proposal, these cases might have affected the quantitative outcomes of our results.

### Future directions

To overcome the gap in treatment information that is provided to patients, personalized pamphlets (infographics) describing treatment options (i.e., curative and palliative options), and their outcomes (i.e., survival, functional, and quality of life outcomes) could be provided directly after diagnosis, informing patients about all treatment options prior to the outpatient clinic consultation for finally deciding on the treatment. Additionally, decision aids have been helpful in facilitating patient engagement in treatment decision‐making and could enhance the patient's question asking behavior.^38^, ^39^, ^40^, ^41^ An ongoing trial (the SOURCE trial: NCT04232735) investigates the effects of personalized predictions of treatment outcomes,^42^ used by caregivers who are trained in using this specific tool on tailored information provision to patients diagnosed with esophagogastric cancer. The outcomes of such studies may improve personalized and tailored information provisions in clinical consultations.

### Conclusion

Since changes in the multidisciplinary team meeting proposal and actual treatment carried out rarely occurred, the multidisciplinary team meeting proposal is pivotal. Differences in the beliefs and personalities of the attending clinicians might have contributed to the variation during the outpatient clinic consultation. Patients who aimed to be cured of their cancer trusted their treating clinician and were less concerned with the harms of a certain treatment.

## CONFLICT OF INTEREST

PS: Research support or funding: EndoStim, Pentax, Norgine, Motus GI and The Enose company Advisory Board: Motus GIE; HvL: Consultant or advisory role: BMS, Lilly, MSD, Nordic Pharma, Servier, Research funding and/or medication supply: Bayer, BMS, Celgene, Janssen, Lilly, Nordic Pharma, Philips, Roche, Servier; RV: received research grants from Roche and Bristol‐Myers Squibb; For the remaining authors no conflict of interest was declared.

## AUTHOR CONTRIBUTIONS

JL: conception, design, analysis, acquisition of data, interpretation of data, drafting manuscript, revising manuscript, final approval, accuracy, and integrity; LB: analysis, interpretation of data, acquisition of data, revising manuscript, final approval, accuracy, and integrity; PV: conception, design, analysis, interpretation of data, revising manuscript, final approval, accuracy, and integrity; AW: interpretation of data, revising manuscript, final approval, accuracy, and integrity; FW: interpretation of data, revising manuscript, final approval, accuracy, and integrity; JH: interpretation of data, revising manuscript, final approval, accuracy, and integrity; SM: interpretation of data, revising manuscript, final approval, accuracy, and integrity; JOH: interpretation of data, revising manuscript, final approval, accuracy, and integrity; MvD: interpretation of data, revising manuscript, final approval, accuracy, and integrity; LT: interpretation of data, revising manuscript, final approval, accuracy, and integrity; MH: interpretation of data, revising manuscript, final approval, accuracy, and integrity; HvL: conception, design, interpretation of data, acquisition of finances, revising manuscript, final approval, accuracy, and integrity; CR: conception, design, interpretation of data, acquisition of finances, revising manuscript, final approval, accuracy, and integrity; PS: conception, design, interpretation of data, acquisition of finances, revising manuscript, final approval, accuracy, and integrity; MW: conception, design, analyses, interpretation of data, acquisition of finances, revising manuscript, final approval, accuracy, and integrity; RV: conception, design, analyses, interpretation of data, acquisition of finances, revising manuscript, final approval, accuracy, and integrity; GN: conception, design, analyses, interpretation of data, acquisition of finances, revising manuscript, final approval, accuracy, and integrity.

## ETHICS APPROVAL

The Medical Research Ethics Committees of the Netherlands confirmed that ethical approval was not required for this study (W.18.166). The participating hospitals approved this study. Written informed consent was obtained from all the participants prior to the interviews and focus groups

## Supporting information


**supinfo**.

## Data Availability

The data underlying this article cannot be shared publicly due to for the privacy of individuals that participated in the study. Data are however available from the authors upon reasonable request and with permission of Netherlands Comprehensive Cancer Organization (IKNL).
