# Supplementary material for: Treatment decision‐making during outpatient clinic visit of patients with esophagogastric cancer. The perspectives of clinicians and patients, a mixed method, multiple case study
Source: Cancer Med. 2022 Feb 15;11(12):2427–44. doi: 10.1002/cam4.4596 (PMC9189462; doi:10.1002/cam4.4596)
Supplement: Supplementary file 1 — supinfo. [file CAM4-11-2427-s001.docx]

**Supplementary 1: Interview guide**

*Introduction of interview*

Initial topic list

*Organizational context*

Considerations in care provided (patients notes as a reminder):

• Organization of clinical pathway

• Patient discussion (multidisciplinary team meeting & with colleagues)

• Are all patients discussed in a multidisciplinary team meeting

*Collaboration in region*

• Logistics

• Financial aspects

• Political problems

• Referral

• Process

• Collaboration between centers

*Treatment decision-making:*

• such as: receival, process and influences on treatment

*Knowledge*

• Physician/ hospital

• Conferences

• Centralization and knowledge drainage

*Physician’s preferences*

• Recent positive or negative experience with sort of treatment

• Observed referral difference between colleagues

*Patient related factors*

• Treatment options

• Shared decision making

• Cases of doubt

• Referral

*Considerations in treatment decisions*

**Supplementary 2: Patient characteristics of patient whom participated during the focus group**

| Patient focus group characteristics | |
| --- | --- |
| Patient demographics | Number or mean (range) |
| Sex |  |
| Male | 16 |
| Female | 1 |
| Mean age | 65 (45-78) |
| Year of diagnosis |  |
| 2019 | 1 |
| 2018 | 13 |
| 2017 | 1 |
| 2015 | 1 |
| 2014 | 1 |
| Diagnosis |  |
| Esophageal cancer | 15 |
| Gastric cancer | 2 |
| Education |  |
| None/basic | 3 |
| Secondary education | 2 |
| Post-secondary college | 4 |
| Bachelor | 4 |
| Master | 1 |
| Unknown | 3 |
| Referral for surgery |  |
| Yes | 9 |
| No | 8 |

**Supplementary 3: Patient focus group guide**

**INTRODUCTION**

Introduction of the VARIATE project

Informed consent

*Part 1: introduction (10 minutes)*

Patient introduction:

• Received diagnosis

• Received treatment

• Involved clinicians

*Part 2: Satisfaction and experience with quality of health care (30 min):*

Satisfaction/ experience regarding:

• Explanation of diagnosis

• Discussed treatment options – including alternative treatment options

• Participation in treatment decision-making

• Consideration

• Expectation

• Experience regarding organization of care

*Part 3 reasons for choosing treatment (30 min):*

Reasons for choosing specific treatment:

• Treatment preferences

• Factors influencing treatment decision-making

• Regret

• Benefits and harms

• Involvement of family members

*To conclude (10 min):*

I would like to conclude this conversation. Are there subjects that haven’t been mentioned yet and you would like to discuss?

• Possibility to ask further questions

• I would like to thank you all for your participation

• Travel expenses

• Filling in the patient background question form

• Contact information is handed over to the participants for further follow up if requested
